# Systemic Anticancer Therapy Details and Dental Adverse Effects in Children

**DOI:** 10.3390/ijerph19116936

**Published:** 2022-06-06

**Authors:** Anna Jodłowska, Lidia Postek-Stefańska

**Affiliations:** Department of Pediatric Dentistry, Faculty of Medical Sciences in Zabrze, Medical University of Silesia, 40-055 Katowice, Poland; lstefanska@sum.edu.pl

**Keywords:** tooth abnormalities, dental development, chemotherapy

## Abstract

An idea of therapy intensification in order to make anticancer treatment more effective is still being investigated. The study aimed to estimate the impact of the chemotherapy dose levels and treatment duration on the risk for dental development disturbance. The clinical examination and OPG analysis were carried out in 37 cancer survivors and germ agenesis, microdontia, size reduction, taurodontism, root and enamel abnormalities were identified. An analysis of anticancer treatment was carried out separately for vincristine (VCR), doxorubicin (DXR), cyclophosphamide (CP), etoposide (VP-16), carboplatin (CBDCA) and actinomycin D (ACTD) recipients in terms of treatment duration and drug doses administered. Individuals aged between three years and ten months, and seven years and four months, at diagnosis presented with no severe dental abnormalities, regardless of treatment duration and increasing cytotoxic drug doses. The largest number of abnormalities per one person was noted in the survivors treated with the highest single doses of VCR, DXR, CP and ACTD. No similar observation was made in the cases of cumulative and weekly doses analyzed. Moreover, there were no significant differences between the mean number of abnormalities across all the drug groups.

## 1. Introduction

Different malignancies usually present with a great spectrum of clinical features and the prognosis determining the choice of treatment regimen. A low-risk disease requires minimal therapy, sometimes limited only to surgery. However, chemotherapy is recommended in most cases, though it may be intensive or of less toxicity depending on the risk associated with the disease. Furthermore, after an intensive protocol is realized and remission is documented, maintenance therapy is additionally often introduced.

Although efforts have been made to reduce the toxicity of anticancer drugs, the idea of intensification of therapy in order to make treatment more effective is still being investigated. It has been proven that more aggressive chemotherapy provides better remission rates, though the risk for acute adverse effects increases [1]. To prevent acute or long-term sequelae, a number of agents, such as granulocyte colony-stimulating factor or mesna, were incorporated into therapy [2,3]. Moreover, methods prolonging the retention time of free vincristine (VCR) in the plasma and, by the same token, decreasing the VCR clearance are investigated to make chemotherapy more effective [4]. Consequently, there are many clinical trials introducing new protocols with shorter treatment intervals or based on higher drug doses [5,6]. 

The problem of dental adverse effects after anticancer therapy has been widely discussed in the literature. Disturbed odontogenesis has been confirmed in numerous clinical research studies [7,8,9,10,11,12,13,14,15,16,17,18]. Moreover, the impact of some anticancer drugs was also illustrated histologically on either the animal model or deciduous human teeth [19,20,21,22,23,24,25]. In one of the authors’ recent papers, it was reported that up to 92% of the total number of abnormal teeth found in the study could have had the injury caused by antineoplastic therapy because the treatment time overlapped with their expected odontogenesis [26]. However, why some teeth are abnormal, and some are properly developed, is still open to debate in the context of the drug dose level, treatment intervals and others. The risk for tooth germ impairment has not been studied in detail in connection with the dose received and treatment duration. In the previous study, the authors of the current paper tried to determine the difference between the affected and non-affected population in six groups of survivors presenting separately with: agenesis, microdontia, tooth size reduction, microdontia and/or tooth size reduction, enamel defects and taurodontism. The above-mentioned differences have been established in terms of the entire treatment duration and cumulative drug doses. No significant differences in the treatment duration were noted between analyzed abnormalities within the affected and non-affected population for each analyzed medication. No significant differences were also established between the affected and non-affected participants within each tooth abnormality group in terms of treatment duration and cumulative drug doses administered in the first 10 and 90 weeks of the therapy. In some cases, treatment duration was even longer in the non-affected group than in the group with analyzed abnormality. Some strong significant correlations were also found between analyzed values; however, no reliable conclusions were established based on these observations [27]. In conclusion, it has not been determined yet whether the intensification of therapy is the reason for more common dental changes. No authors to date have addressed the problem. Only cumulative drug doses were studied [7,27,28]. On the other hand, owing to the fact that developing dental tissues are highly susceptible to toxic impairment, similarly to cancer cells, even a small dose of the drug could cause irreversible changes in odontogenesis. Therefore, maintenance therapy is also likely to be very toxic for rapidly dividing dental cells or dental blast cells.

The study aimed to estimate the impact of the chemotherapy dose levels and treatment duration on the risk for dental development disturbance.

## 2. Material and Methods

The observational study was approved by the Bioethics Committee at the Medical University of Silesia in Katowice, Poland, on 25 February 2013 and on 29 November 2016 (KNW/0022/KB1/15/I/13, KNW/0022/KB1/15/II/16). The main assumption of the cross-sectional study was to enroll cancer survivors who fulfilled the following inclusion criteria: anticancer therapy started before 10 years of age and completed at least two years before dental examination. A total of 37 cancer survivors aged 6–17 years were subjected to careful clinical and radiological dental examinations in the Outpatient Clinic of Pediatric Dentistry, after the caregivers gave their written informed consent for participation in the study and further publication. Following the clinical examination and OPG analysis, dental developmental abnormalities were diagnosed and divided into groups with size anomalies (S) and enamel disturbances (E), according to the assumption of a different coming out time during tooth formation and based on observations that there are many other environmental factors influencing the occurrence of enamel changes. Germ agenesis (A), microdontia (M), size reduction (R), taurodontism (T) and root abnormalities (Rt) were identified as size problems. Among enamel anomalies, the authors found opacities (O), deep perikymata (P) and hypoplasia (H). The group’s characteristics including anticancer treatment details are summarized in Table 1.

An analysis of anticancer treatment was carried out separately for VCR, doxorubicin (DXR), cyclophosphamide (CP), etoposide (VP-16), carboplatin (CBDCA) and actinomycin D (ACTD) recipients in terms of treatment duration and drug doses administered. Special attention was paid to the duration of intensive and maintenance therapies with all drugs administered, and the duration of treatment with each analyzed drug for the entire period of therapy. When it comes to the dose level, single and cumulative drug doses were identified for intensive and maintenance therapy, as applicable, to determine an average weekly dose for each drug. The single dose level of chemotherapy predominantly increased with the patient’s age in each drug group, which is why an average of a single dose was not calculated. A comparative analysis of the mean values concerning therapy duration and cumulative drug doses was carried out with reference to the mean number of abnormalities.

The majority of analyzed drugs was scheduled during intensive protocols; therefore, the tables with more detailed treatment data are not included in the study. Consequently, only in some VCR and CBDCA patients, who were receiving anticancer drugs throughout the entire therapy, a comparative statistical analysis between intensive and maintenance therapy was performed.

Another step was to assess the impact of dose levels on the occurrence of developmental disturbances. The single, weekly and cumulative doses were divided into three equal intervals: lowest (I), medium (II) and highest (III). After that, a comparative study was conducted separately for size (S) and all abnormalities (S + E). Thus, the following null hypothesis was formulated—if approach to dose calculation does not matter, then the number of reported abnormalities should be similar when comparing these dose representations. Therefore, a statistical analysis was introduced to compare abnormalities’ distribution in patients receiving each drug at different dose levels: I dose range, II dose range and III dose range calculated using different approaches (single dose vs. weekly dose vs. cumulative dose). Distributions within each subgroup were calculated as sums of abnormalities in these groups (e.g., number of all reported S abnormalities in patients receiving I dose range calculated on the basis of complete dosage).

Additionally, the treatment details related to administration frequency were collected and taken into consideration.

### Statistical Analysis

In the study, quantitative features were assessed. In order to characterize the structure of the studied variables, basic descriptive statistics in the form of measures of position and variability were calculated. The differences between drug groups in terms of the number of size abnormalities and number of enamel abnormalities were identified using a non-parametric Kruskal–Wallis test by rank. The U Mann–Whitney test was used to compare differences between two groups of abnormalities. The Shapiro–Wilk test was adopted to verify the normality of the distribution. In order to verify the significance of differences between the results in intensive and maintenance therapies, the Student’s *t*-test was used for dependent samples and its non-parametric counterpart in the form of the Wilcoxon paired test was also applied. To compare abnormalities’ distribution in patients receiving each drug at different dose levels calculated using different approaches, a chi-square test was used. The Fisher’s test was applied for groups of less than 5 individuals. A value *p* ≤ 0.05 was considered as statistically significant. All statistical calculations were performed with the use of Statistica Version 13.3 software (StatSoft Polska, Krakow, Poland).

## 3. Results

In the study group, the mean duration of treatment was 59.73 weeks, varying from 5 weeks minimum to 122 weeks maximum. In 15 patients, the entire anticancer therapy was planned as an intensive treatment regimen ranging between 5 and 78 weeks (Table 1). Table 2 presents the mean values of treatment duration with all and six analyzed drugs, and the mean cumulative doses during intensive and maintenance therapies for each drug group. No significant differences between the mean number of size abnormalities and enamel abnormalities across all the drug groups were found. The mean number of size abnormalities was statistically significantly higher than the mean number of enamel abnormalities within almost all drug groups (*p* < 0.05) except ACTD (*p* = 0.36).

In the VCR and CBDCA groups, some individuals received the analyzed drug during the entire therapy. The results of the analysis of medical records with quantitative data regarding the number of dental abnormalities for both drug groups are included in Table 3 and Table 4, respectively. In terms of treatment duration, the intensive therapy was found to be statistically significantly shorter relative to the maintenance treatment in both analyzed groups: VCR (*p* = 0.014), CBDCA (*p* = 0.028). No statistically significant difference between the mean cumulative VCR doses administered in the intensive and maintenance periods of treatment was determined (*p* = 0.72); however, the mean VCR weekly dose was statistically significantly higher in intensive therapy (*p* = 0.05) (Table 3). In the case of CBDCA, the mean weekly dose was also statistically significantly higher in the intensive therapy in comparison with maintenance treatment (*p* = 0.0047), although the mean cumulative dose administered during the intensive treatment was statistically significantly lower (*p* = 0.0012) (Table 4).

**Figure 1:**
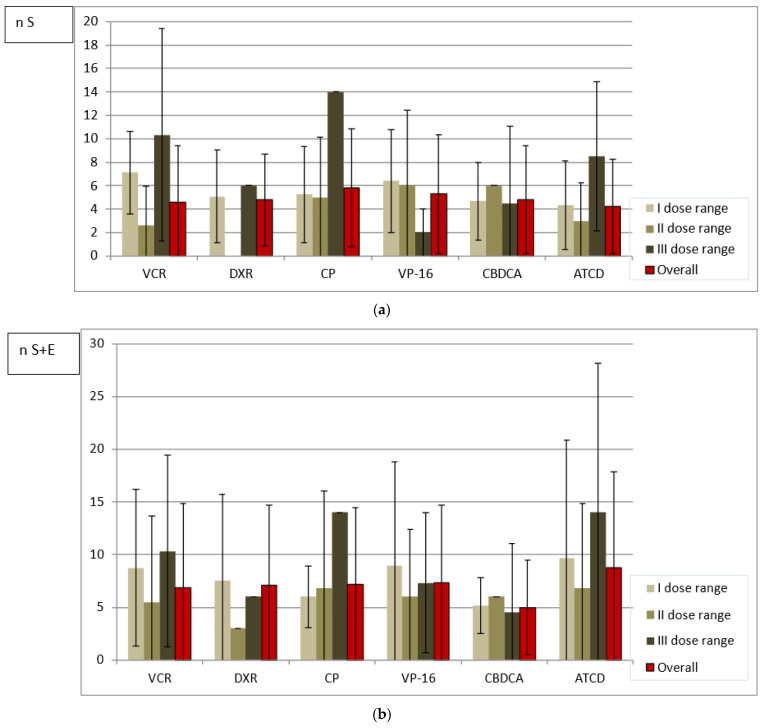
(**a**) Mean number of size abnormalities (n S) in relation to three single dose ranges of analyzed drugs. (**b**) Mean number of all abnormalities (n S + E) in relation to three single dose ranges of analyzed drugs.

**Figure 2:**
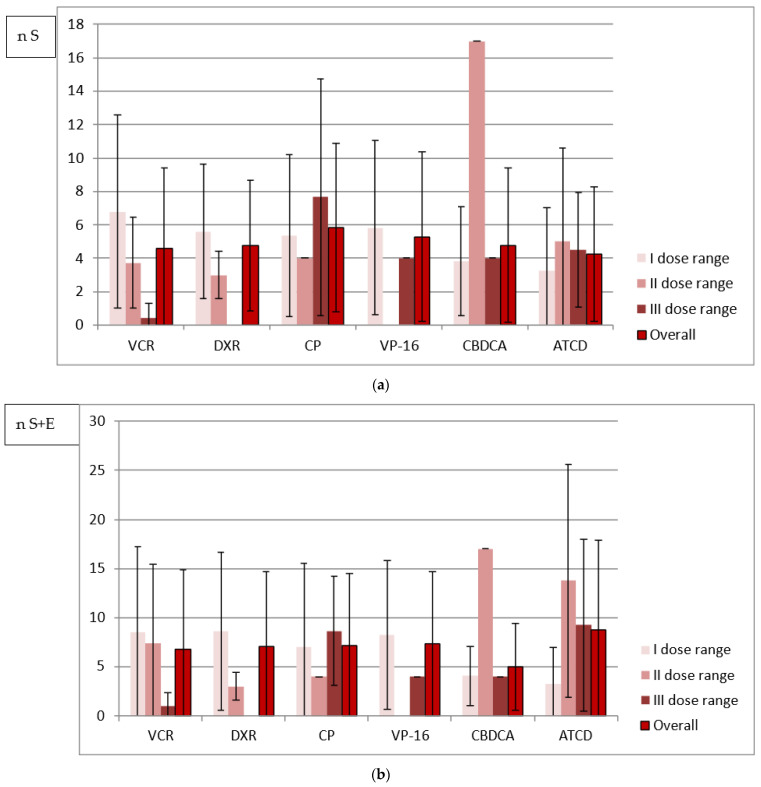
(**a**) Mean number of size abnormalities (n S) in relation to three weekly dose ranges of analyzed drugs. (**b**) Mean number of all abnormalities (n S + E) in relation to three weekly dose ranges of analyzed drugs.

**Figure 3:**
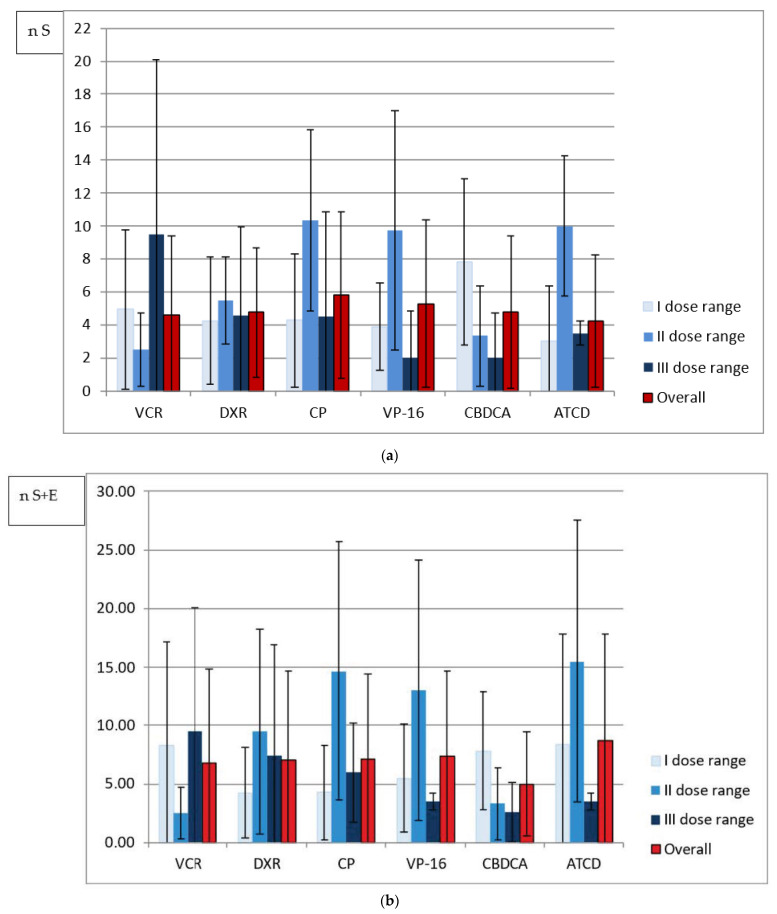
(**a**) Mean number of size abnormalities (n S) in relation to three cumulative dose ranges of analyzed drugs. (**b**) Mean number of all abnormalities (n S + E) in relation to three cumulative dose ranges of analyzed drugs.

In terms of the single dose received, the largest number of either size or all abnormalities per one person was noted in the survivors treated with the highest doses of VCR, DXR, CP and ACTD (an exception: DXR-all abnormalities). No similar observation was made in cases of cumulative and weekly doses analyzed because the largest number of abnormalities was diagnosed after administration of the highest drug doses only in patients from the VCR and CP groups, respectively. However, in the majority of patients who received the least toxic drug doses, regardless of the method of analysis, there was a higher or comparable number of tooth anomalies in relation to the mean number calculated separately for each drug group (Figure 1, Figure 2 and Figure 3).

A statistical analysis, introduced to compare abnormalities’ distribution in patients receiving particular drugs at different dose levels calculated using various approaches, revealed a dependency between these factors and the total number of tooth abnormalities in almost all cases (the exception was the results for size abnormalities in CP recipients). It means that the null hypothesis can be rejected, and the distribution of abnormalities at different dose levels differs between dose calculation approaches (Table 5).

When it comes to the duration of treatment intervals, the mean values were established for each drug group, and the characteristics of particular drug administration in anticancer protocols were additionally listed in Table 6. VCR was usually administered in a one-day-cycle in the shortest one-week-intervals, but the shortest mean value for the entire group was established in VP-16 recipients. Relatively long intervals during intensive therapy were observed in the DXR, CP and ACTD groups, whereas during the maintenance treatment period, a similar length of intervals was noticed.

## 4. Discussion

There is strong evidence that the analyzed dental abnormalities are more prevalent in individuals after chemotherapy compared to the healthy generation. Disturbed odontogenesis has been demonstrated after the administration of numerous chemotherapeutic agents interfering with the mitotic cycle of cancer cells [9,10,29,30,31]. This is why that problem is not taken into consideration in the current study. A number of factors increasing the risk for dental abnormalities such as the age at diagnosis, stage of dental development or type and duration of chemotherapy are still being evaluated [11,12,26,32]. Thorough analyses of treatment details taking into account also the age at diagnosis and duration of the entire therapy are uncommon in the literature. No efforts have been made for an in-depth analysis of individual and cumulative drug doses, treatment intervals in combination with the length of chemotherapy, age at treatment and the number of dental anomalies. In the current study, six drugs were evaluated because of the most frequent use (Table 2). In the CP drug group, the highest mean number of affected teeth was noted. The whole CP regimen was completed in the intensive part of treatment and was the longest among all drug groups. However, it should be noted that the CP patients also had the longest mean duration of intensive and entire therapy with all drugs. According to Ramirez et al. the human metabolism exhibits a lower rate of CP pharmacokinetics compared to dogs, cats or mice [33]. CP administration in leukemia protocols was realized in long intervals of one-day-cycles (6–9 weeks). In some regimens dedicated for solid tumor therapy, three-day-cycles of CP were sometimes administered, and the intervals were very long (6–10 weeks in nephroblastoma treatment) (Table 6). Hsieh et al. reported that children who received high doses of CP were at an increased risk for dental disturbances. No similar observations referring to the analyzed VCR, DXR, ACTD and vinblastine have been made [34]. Kaste et al. revealed a dose-dependent risk of having at least one dental abnormality in the group of survivors younger than five years old treated with alkylating agents in comparison to those who received no alkylating agents [28]. In another study, treatment with CP, DXR and ifosfamide (IF) and their doses, related to hypodontia, microdontia and root resorption, and enamel defects, had a strongly negative impact on odontogenesis. A positive correlation between the absence of tooth buds and the administration of VCR, CP, DXR, IF and VP-16 was also noted. Moreover, a positive correlation was established between microdontia and treatment with VCR, DXR, CP, IF, VP-16, cisplatin (CDDP) and 5-fluorouracil. VP-16 and CDDP treatments were related to microdontic teeth, root resorption and enamel defects. A positive correlation between taurodontic teeth and VCR administration was also identified [32].

In the present study, the smallest number of teeth abnormalities was found in the ACTD group, despite the relatively low age of anticancer drug administration. However, the ACTD group presented with the shortest treatment duration with ACTD of approximately 14 weeks, but not the shortest intensive anticancer protocol (32.92 weeks). In relation to the fact that there were no significant differences between the mean number of abnormalities across all the drug groups, no significant differences in terms of treatment duration were evaluated (Table 2). Moreover, the treatment with a particular drug overlaps with the entire therapy, and determining the impact of treatment duration for an individual agent seems to be impossible. In the current study, the longest treatment protocols were administered to leukemia survivors. Intensive treatment lasted 30–42 weeks, whereas entire therapy ranged from 104 to 122 weeks. The younger patients, aged two years and five months at diagnosis, had expected dental changes. They had permanent second premolars and second molars reduced in size, respectively, to the developmental dental sensitive period. Long maintenance therapy does not seem to be a significant factor for the higher risk for dental changes if the treatment started at two years and five months. On the contrary, older leukemia survivors started their treatment at the age of approximately four years and presented with no abnormal teeth. They received their anticancer treatment in the period which is not considered critical, and even though the therapy was very long, no noticeable dental changes were noted. The phenomenon was already addressed in one of the authors’ previous papers [26]. In another study by the author, no significant differences in therapy duration were found between the affected and non-affected survivors within almost all groups of dental abnormalities for each drug. An exception was established for microdontia in the DXR group (*p* = 0.04), with the therapy longer in affected survivors and a reduction in crown size in patients treated with CP (*p* = 0.03), with a paradoxically longer treatment duration in non-affected participants [27]. Proc et al. also demonstrated that the distribution of microdontic teeth depended on the age during anticancer treatment [9]. Kang et al. made an observation that agenesis and microdontia were the most prevalent in younger survivors, while root abnormalities increased with the age at treatment [12]. Treatment duration also did not contribute to the occurrence of dental sequelae in the patients treated for nephroblastoma, but the homogeneity of the group could influence the outcome [13]. These observations are in opposition to the intuitive belief that the longer therapy is administered, the more probable its toxic impact. Maguire et al. pointed to long leukemia therapy as a probable cause of severe opacities and hypoplasia [8]. Krasuska et al. revealed that the longer the antineoplastic therapy was, the more frequent and severe the dental abnormalities were. Every congenital abnormality was strongly correlated to the duration of antineoplastic treatment [32]. Oguz et al. assessed a small homogenous cohort, with treatment duration not considered in this study, and did not find any correlation between dental developmental changes and patient age at diagnosis [18]. No significant changes in the number of affected teeth between the age groups were also reported in heterogeneous survivors, but the treatment duration was not analyzed either [14].

Due to the weight-dependency of drug administration, it is easy to observe that in most cases younger patients received smaller doses compared to the older individuals with sometimes more than two-fold single doses (Table 3 and Table 4). Even so, antineoplastic therapy affects younger patients more severely, as previously demonstrated [7,8,9,10,12,14,15,16,17]. The present study confirms this observation. Consequently, even a small dose of a cytotoxic agent in multidrug therapy is likely to be sufficient to impair dental tissues as long as treatment is provided in a susceptible stage of their formation. Moreover, short therapy duration is likely to be safer for developing cells. In the analyzed study group, two young patients diagnosed with nephroblastoma at the age of 23 and 25 months had a relatively short entire therapy of 11 months. One survivor had no abnormalities, even though VCR was administered every week, and the other one presented only with enamel white spots involving the permanent first molars. An exceptionally short therapy duration of five weeks was noted in a patient aged three years and ten months at diagnosis, and he had no abnormal teeth as well. However, in our study, individuals aged between three years and ten months and seven years and four months at diagnosis presented with no severe dental abnormalities, regardless of increasing cytotoxic drug doses. If the female patient aged three years and five months, who had four second premolars missing and late dental eruption in the history, was excluded from the observation, the lower age limit would decrease to three years and one month in the current study. The conclusion is that the above-mentioned age interval does not seem to be a developmentally critical period for dental tissues. This is clearly shown in Table 3 and Table 4. It is also in accordance with some papers found in the literature [9,26]. Thanks to observational studies on cancer survivors, the adverse effects of all environmental factors influencing dental development may not be expected in the preschool stage in children.

The majority of regimens are based on a combination of different drugs during intensive and maintenance therapies, which makes it difficult to evaluate the toxicity of both treatment protocols. Taking into account mean drug doses, a mean cumulative dose received during the entire therapy was in some cases even twice as high as the cumulative dose administered during intensive treatment (Table 2). In the VCR and CBDCA groups, the maintenance therapy was almost twice as long as the intensive protocol; therefore, the patients received a smaller dose of the drug in the unit of maintenance treatment time. VCR was one of the analyzed agents often used during the entire therapy. The data of 11 survivors were distinguished in order to compare either the drug doses or treatment intervals. In almost all cases presented in Table 3, the single dose did not change during maintenance therapy compared to the intensive protocol. However, in the majority of cases, the VCR doses received per week were smaller during the second treatment period thanks to the longer treatment intervals (Table 3). In one patient, VCR was administered during the intensive protocol, but it was replaced with vinblastine during maintenance therapy, and the treatment intervals were short during the entire 88-week therapy. The patient presented with an exceptionally high number of abnormal teeth despite a relatively late cancer diagnosis. Conversely, two similarly aged (nine months and eleven months) participants had VCR treatment of 36 and 39 weeks’ duration, respectively, and 24 and 11 drug doses were administered to them, respectively. However, the older one had 26 affected teeth diagnosed in comparison with 7 affected teeth found in the younger participant, even though the single doses were smaller and the treatment intervals longer. It is necessary to consider the duration of the entire multiagent therapy. In the older participant, the anticancer treatment was twice as long, and this factor was probably more decisive for the occurrence of adverse effects (Table 3). In the CBDCA group, with the same single drug dose throughout the entire therapy, a mean cumulative CBDCA dose was more than 2.5 times higher during more than 3.5 times longer maintenance treatment in relation to the intensive phase. Interestingly, one can observe only a 1.5-fold decrease of the weekly dose. Nevertheless, children aged three years and ten months and older had no dental side effects diagnosed despite similar therapy duration and single drug dose increasing with age (Table 4).

In the current study, the children receiving VCR, CP and VP-16, and the majority of individuals receiving CBDCA and ACTD were treated with single doses increasing with age. Conversely, drug administration in the DXR group and sometimes in the CBDCA and ACTD groups varied depending on weight, diagnosis and anticancer regimen used (data available upon request). The dose level division is likely to be more reliable for the study assessment. A statistical analysis of the distribution of the abnormalities number within drug groups revealed that it differs depending on the dose calculation approach in almost all cases. Only in CP recipients with size abnormalities (S) does the comparison between single, weekly and cumulative doses not allow one to say that the distribution of abnormalities differs when dose range is calculated using different approaches (*p* > 0.05) (Table 5). Based on the data shown in Figure 1, Figure 2 and Figure 3, in the majority of patients who received the lowest drug doses, regardless of the method of analysis, a higher or comparable number of tooth anomalies in relation to the mean number calculated separately for each drug group was diagnosed. This could suggest that even a small dose of a toxic drug can cause tooth development impairment. It must also be highlighted that the level of the single dose is dependent on the body weight or surface. The lowest doses are usually received by the youngest patients with the highest susceptibility to the toxic effects of therapeutic agents. However, the study survivors treated with the highest single VCR, CP and ACTD doses presented with exceptionally high number of abnormalities (Figure 1). While the CP recipients were affected more severely, as already mentioned, the children who received VCR and ACTD had a relatively smaller mean number of dental abnormalities (Table 2). It may be that the estimated weekly dose will be a better expression of the actual drug dose received. In the VCR, DXR and VP-16 groups with the lowest drug range, the survivors had the highest number of abnormalities per one person. In the CBDCA and ACTD groups with the higher weekly dose, a greater number of tooth abnormalities was noted. However, it is difficult to say that CP could have the lowest toxicity based on the weekly dose assessment in this study. In the patients who received the lowest weekly CP doses, the number of abnormalities was very close to the mean and, in the group with the highest weekly doses, the biggest number of anomalies was documented (Figure 2). An analysis of cumulative drug doses is likely to be less reliable. It is difficult to recognize that medium drug doses are more toxic in comparison with high cumulative doses used for chemotherapy (Figure 3). The small study cohort is not the only research limitation, but either multidrug character of the anticancer therapy. An analysis of cumulative drug doses, very often found in the literature, gives no information of the single dose level or the drug administration frequency. In the current study, the irregularity of the results of the cumulative dose analysis seems to confirm that it does not matter how high the total drug level is. Despite the fact that no statistically significant difference between the mean cumulative VCR doses administered in the intensive and maintenance periods of treatment was determined (*p* = 0.72), and the mean cumulative CBDCA dose was even statistically significantly lower during intensive treatment (*p* = 0.0012), the mean weekly dose was statistically significantly higher in the statistically significantly shorter intensive therapy in both drug groups (Table 3 and Table 4). The different results presented in the analyses of Figure 1, Figure 2 and Figure 3 are likely to contradict an assumption in research based on the toxic impact of drug dose on dental development: that the higher the drug dose administered, the more common the tooth abnormalities. This is in line with the foregoing observation that the most probable factor determining the occurrence of the dental abnormalities is likely to be the stage of tooth development during anticancer treatment, i.e., the patient’s age. Owing to the fact that anticancer therapy has a predominantly multidrug character, a large study cohort and similarly detailed analysis is needed.

The intensification of chemotherapy can make antineoplastic treatment more effective and improve prognosis. This can be achieved by using a higher dose of the drug, or the more frequently used practice of decreasing the duration of intervals [2,5,19,35,36]. Although the anticancer agent remains toxic to the dividing tissue over a short time, repeated administration can make regeneration of dental immature cells impossible. There are no assessments of how often the drug was administered and how long the treatment intervals were in the context of dental development. In addition, there is no information on whether short treatment intervals are more aggressive for developing dental tissues, as could be expected. It is worth taking into account the fact that the most severely affected was a patient treated for nephroblastoma, in whom two third molars did not develop, and four second premolars and three second molars appeared microdontic. He received a 39-week therapy with 12 cycles given every three weeks when he was two years and four months old. CP combined with DXR and VP-16 with CBDCA were used in a staggered manner in a three-day-cycle. His age-mate received the same treatment protocol preceded by a six-week-administration of VCR, ACTD and DXR combined. He presented with agenesis of two second molars and microdontia of the remaining second molars. Longer therapy affected only one group of teeth, but the effect was more severe. This interesting analysis may explain the unknown facts about dental development if a study is conducted on a larger cohort. When it comes to drug administration, three-week-intervals are likely to be safer for undifferentiated cells with extraordinary reproducing ability. Nevertheless, in the analyzed survivors, the most severe abnormalities were diagnosed. The VCR group exposed to chemotherapy predominantly scheduled in one-week- and rarely in three-week-intervals did not have an outstanding number of teeth affected. By contrast, CP, described in the literature as the most aggressive cytotoxic agent, was administered with the longest mean interval and only three times during the whole therapy in leukemia regimens, sometimes with 13 weeks between each administration. VP-16 was observed as having the shortest mean treatment intervals and a relatively high number of abnormal teeth and medium therapy duration. A thorough analysis revealed that VP-16 was scheduled in the longest 3–5-day-cycles, which may be a decisive risk factor (Table 6). Krasuska et al. found a positive correlation between the absence of tooth buds and the use of VP-16. VP-16 was also related to microdontic teeth, root resorption and enamel defects. However, Krasuska et al. also noted that in the literature VP-16 was not considered risky for dental development [32].

### Limitations

There are certain limitations associated with the study. A small study cohort with different multiagent treatment protocols realized, various ages at diagnosis and different treatment durations may have an impact on the outcome of the research. Moreover, many cytotoxic drugs are often replaced with their different analogs during the therapy of one patient, which always interferes with the study inclusion criteria.

## 5. Conclusions

In the present study, no evidence was found for the particular drug effect of treatment duration and cumulative drug dose received on the frequency of dental developmental abnormalities. Individuals aged between three years and ten months and seven years and four months at diagnosis presented with no severe dental abnormalities, regardless of increasing cytotoxic drug doses. The largest number of abnormalities per one person was noted in the survivors treated with the highest single doses of VCR, DXR, CP and ACTD. However, no significant differences were shown between the drug groups in terms of the mean number of teeth affected. Moreover, with respect to the different treatment protocols used and the small sample size, research based on a more homogenous group of survivors seems necessary.

## Figures and Tables

**Table 1:** Baseline characteristics of the study group.

		Number of Survivors
**Age at cancer diagnosis**	0–3	21
	3, 1–5	10
	5, 1–9	6
**Type of cancer diagnosis**	Solid tumors*	28 (1 survivor with subsequent leukemia)
	Hematological cancers*	9
**Type of treatment**	Surgery	26
	Radiotherapy	12 (head radiotherapy in 4 participants)
	Chemotherapy	37
		**Mean duration of the therapy (weeks)**
**Type of the therapy with all drugs**	Intensive treatment	26 (5 minimum/78 maximum)
	Entire therapy	59.73 (5 minimum/122 maximum)
		**Number of teeth affected**
**Dental abnormalities in the study group**	Size abnormalities	Agenesis—20 Microdontia—30, Reduction in size (microdontia excluded)—59 Taurodontism—27 Root abnormalities—27
	Enamel abnormalities	Opacities—40 Deep perikymata—16 Hypoplasia—24
		**Number of survivors treated with particular drug (100%)/Number of survivors with dental abnormalities**
**Drug analyzed**	VCR	30/22 (73.33%)
	DXR	13/11 (84.62%)
	CP	12/10 (83.33%)
	VP-16	14/12 (85.71%)
	CBDCA	14/11 (78.57%)
	ACTD	12/10 (83.33%)

Solid tumors* diagnosed in the study participants: nephroblastoma, neuroblastoma, medulloblastoma, rhabdomyosarcoma, hepatoblastoma, anaplastic ependymoma, infantile fibrosarcoma, sarcoma granulocyticum, teratoma malignum, embryonal primitive neuroectodermal tumor (PNET)/Ewing sarcoma (ES), yolk sac tumor, clear cell sarcoma, astrocytoma pilocyticum; Hematological cancers* found in the study participants: acute lymphoblastic leukemia, Hodgkin lymphoma, myelomonocytic lymphoma; VCR—vincristine; DXR—doxorubicin; CP—cyclophosphamide; VP-16—etoposide; CBDCA—carboplatin; ACTD—actinomycin D.

**Table 2:** Treatment duration and drug doses in relation to number of teeth affected and age at diagnosis.

	Mean Values							
Drug Group	Intensive Therapy Duration (All Drugs)	Entire Therapy Duration (All Drugs)	Duration of the Treatment with Particular Drug during Intensive Therapy	Duration of the Treatment with Particular Drug during Maintenance Therapy	Cumulative Dose of the Drug during Intensive Therapy	Cumulative Dose of the Drug during Entire Therapy	Number of Teeth Affected A, M, R, T, Rt/O, P, H **	Age of the Group at Treatment Onset
	Weeks SD	Weeks SD	Weeks SD	Weeks SD	mg (VCR, DXR, CP, VP-16, CBDCA) µg (ACTD) SD	mg (VCR, DXR, CP, VP-16, CBDCA) µg (ACTD) SD	n/n *p* Value ***	Months Min/Max
VCR	27.27 ±17.11	63.87 ±32.77	19.33 ±11.20	35.33 ±11.05	8.45 ±3.96	12.25 ±8.16	4.60/2.23 *p* = 0.027	36.43 4/91
DXR	32.38 ±15.43	49.38 ±21.64	24.85 ±14.21	X	157.43 ±73.40	157.43 ±73.40	4.77/2.31 *p* = 0.043	29.85 4/72
CP	40.17 ±13.93	82.42 ±33.60	28.33 ±6.36	X	2751.04 ±1171.05	2751.04 ±1171.05	5.83/1.33 *p* = 0.014	36.75 11/91
VP-16	31.21 ±18.91	50.86 ±23.96	20.71 ±13.14	13.00 *	1104.11 ±742.99	1155.54 ±683.20	5.29/2.07 *p* = 0.020	35.43 4/102
CBDCA	24.43 ±15.38	56.00 ±18.37	16.43 ±13.02	31.43 ±10.13	1476.04 ±971.48	2968.46 ±1855.58	4.79/0.21 *p* = 0.002	36.57 4/88
ACTD	32.92 ±14.95	42.17 ±20.80	14.75 ±12.29	13.00 *	2991.92 ±2300.63	3316.92 ±2077.53	4.25/4.50 *p* = 0.36	27.33 9/55

Drug dose unit: mg (VCR, DXR, CP, VP-16, CBDCA); µg (ACTD); X—no maintenance therapy; *—no statistically significant number of patients; A—agenesis, M—microdontia, R—crown reduction in size, T—taurodontism, Rt—root abnormalities, O—opacities, P—perikymata, H—hypoplasia; ** Kruskal–Wallis test—no statistically significant differences between drug groups in terms of number of size abnormalities and number of enamel abnormalities; *** U Mann–Whitney test—differences between number of size and number of enamel abnormalities within the drug group.

**Table 3:** Therapy characteristics in patients receiving VCR during intensive and maintenance treatment in relation to number of teeth affected and type of anomaly.

Age	Entire Therapy Duration (All Drugs)		Cumulative Dose of VCR during the Therapy		VCR Treatment Details during Intensive Therapy				VCR Treatment Details during Maintenance Therapy				Number of Teeth Affected	Type of Tooth Anomaly
	Intensive	Maintenance	Intensive	Maintenance	Single Dose	Number of Doses	Therapy Duration	Mean Weekly Dose	Single Dose	Number of Doses	Therapy Duration	Mean Weekly Dose	A, M, R, T, Rt/O, P, H	
Months	Weeks	Weeks	mg	mg	mg		Weeks	mg	mg		Weeks	mg		
4	49	33	5.83	9.45	0.53	12	28	0.21	0.68	14	31	0.3	3/0	R
15	10	41	7.2	5.76	0.72	10	10	0.72	0.72	10	38	0.15	6/0	M, R, T
18	32	18	7.5	3.75	0.75	10	32	0.23	0.75	5	8	0.46	6/0	M, R, T
21	15	72	10.78	10.5	0.98	11	15	0.72	1.05	10	37	0.28	5/0	R, T
26	10	38	8.1	9	0.9	9	10	0.81	0.9	10	33	0.27	5/0	R
37	19	37	5.4	6.3	0.9	6	19	0.28	0.9	7	25	0.25		
46	9	41	8.78	9.75	0.98	9	9	0.98	0.98	10	33	0.3		
60	10	77	11	4	1.1	10	10	1.1	1.1–1.2	13	49	0.3		
69	10	39	12	9.6	1.2	10	10	1.2	1.2	10	37	0.26		
72	10	56	7.2	25.2	1.2	6	7	1.03	1.2	21	41	0.61	2/0	T
88	11	62	2.7	32.4	1.35	2	11	0.25	1.35	24	46	0.7	17/0	M, Rt
Mean	16.81	46.73	7.86	11.43		8.64	14.64	0.68		12.18	34.36	0.35		
			*p* = 0.72 *				*p* = 0.014 *					*p* = 0.05 *		

A—agenesis, M—microdontia, R—crown reduction in size, T—taurodontism, Rt—root abnormalities, O—opacities, P—perikymata, H—hypoplasia; * Wilcoxon paired test.

**Table 4:** Therapy characteristics in patients receiving CBDCA during intensive and maintenance treatment in relation to number of teeth affected and type of anomaly.

Age	Entire Therapy Duration (All Drugs)		Cumulative Dose of CBDCA during the Therapy		CBDCA Treatment Details during Intensive Therapy				CBDCA Treatment Details during Maintenance Therapy				Number of Teeth Affected	Type of Tooth Anomaly
	Intensive	Maintenance	Intensive	Maintenance	Dose	Number of Doses	Therapy Duration	Mean Weekly Dose	Dose	Number of Doses	Therapy Duration	Mean Weekly Dose	A, M, R, T, Rt/O, P, H	
Months	Weeks	Week	mg	mg	mg		Weeks	mg	mg		Weeks	mg		
15	10	41	1056	2640	264	4	10	105.6	264	10	38	69.47	6/0	M, R, T
21	15	72	1787.5	3850	357.5	5	15	119.17	385	10	37	104.05	5/0	R, T
26	10	38	990	3300	330	3	8	123.75	330	10	37	89.19	5/0	R
46	9	41	715	3575	357.5	2	4	178.75	357.5	10	37	96.62		
60	10	77	1606	2409	401.5	4	10	160.6	401.5	6	21	114.71		
69	10	39	1760	4400	440	4	10	176	440	10	37	118.92		
Mean	10.67	51.33	1319.08	3362.33		3.67	9.5	143.98		9.33	34.5	98.83		
			*p* = 0.0012 **				*p* = 0.028 *							
								*p* = 0.0047 **						

A—agenesis, M—microdontia, R—crown reduction in size, T—taurodontism, Rt—root abnormalities, O—opacities, P—perikymata, H—hypoplasia; * Wilcoxon paired test; ** *t*-Student test.

**Table 5:** Number of reported abnormalities depending on dose range and dose calculation approach—chi-square test results for single dose vs. weekly dose vs. cumulative dose.

n S				n S + E			
VCR	*p* < 0.001 *			VCR	*p* < 0.001 *		
	I	II	III		I	II	III
Single	57	50	31	Single	70	104	31
Weekly	95	41	2	Weekly	119	81	5
Cumulative	99	20	19	Cumulative	166	20	19
DXR	*p* < 0.001 *			DXR	*p* < 0.001 *		
	I	II	III		I	II	III
Single	56	0	6	Single	83	3	6
Weekly	56	6	0	Weekly	86	6	0
Cumulative	17	22	23	Cumulative	17	38	37
CP	*p* = 0.285			CP	*p* < 0.001 *		
	I	II	III		I	II	III
Single	21	35	14	Single	24	48	14
Weekly	24	48	14	Weekly	56	4	26
Cumulative	30	31	9	Cumulative	30	44	12
VP-16	*p* < 0.001 *			VP-16	*p* < 0.001 *		
	I	II	III		I	II	III
Single	32	36	6	Single	45	36	22
Weekly	70	0	4	Weekly	99	0	4
Cumulative	31	39	4	Cumulative	44	52	7
CBDCA	*p* = 0.001 *			CBDCA	*p* < 0.001 *		
	I	II	III		I	II	III
Single	28	12	27	Single	31	12	27
Weekly	46	4	17	Weekly	49	17	4
Cumulative	47	10	10	Cumulative	47	10	13
ACTD	*p* = 0.038 *			ACTD	*p* < 0.001 *		
	I	II	III		I	II	III
Single	13	21	17	Single	29	48	28
Weekly	13	20	18	Weekly	13	55	37
Cumulative	24	20	7	Cumulative	67	31	7

I, II, III—drug dose ranges: lowest range, medium range, highest range, respectively; n S—total number of size abnormalities for I, II or III drug dose range and for one analyzed drug; n S + E—total number of all abnormalities for I, II or III drug dose range and for one analyzed drug, * *p* ≤ 0.05.

**Table 6:** Drugs’ administration characteristics.

Drug	Mean Values Concerning Intensive Therapy			Mean Values Concerning Maintenance Therapy			Data Obtained from Medical Records	
	Number of Doses	Therapy Duration	Intervals’ Duration	Number of Doses	Therapy Duration	Intervals Duration	Cycle Duration	Intervals between Cycles
	Min/Max	Weeks Min/Max	Weeks	Min/Max	Weeks Min/Max	Weeks	Days	Weeks
VCR	9.37 2/24	19.33 5/39	2.06	11.58 5/24	35.33 8/49	3.05	1–2	1–4
DXR	6.77 1/18	24.85 1/47	3.67	-	-	-	1–3	3–13
CP	7.25 3/18	28.33 20/40	3.91	-	-	-	1–3	1–19
VP-16	15.43 0/33	20.71 0/39	1.34	12 * 12/12	13 * 13/13	1.08 *	3–5	3–9
CBDCA	8.29 0/24	16.43 0/38	1.98	9.71 6/12	31.43 13/38	3.24	1–4	3–9
ACTD	4.83 0/13	14.75 0/35	3.05	4 * 1/7	13.00 * 1/25	3.25 *	1–3	2–17

* no statistically significant number of patients.

## Data Availability

All data are included in the study in the form of mean values. Detailed information is available on request from the authors.
